# Lessons Learned Through Two Phases of Developing and Implementing a Technology Supporting Integrated Care: Case Study

**DOI:** 10.2196/34899

**Published:** 2022-04-11

**Authors:** Stephanie Di Pelino, Larkin Lamarche, Tracey Carr, Julie Datta, Jessica Gaber, Doug Oliver, Jay Gallagher, Steven Dragos, David Price, Dee Mangin

**Affiliations:** 1 Department of Family Medicine McMaster University Hamilton, ON Canada

**Keywords:** integrated care, information and communication technology (ICT), program evaluation, older adults, primary care

## Abstract

**Background:**

As health care becomes more fragmented, it is even more important to focus on the provision of integrated, coordinated care between health and social care systems. With the aging population, this coordination is even more vital. Information and communication technology (ICT) can support integrated care if the form of technology follows and supports functional integration. Health TAPESTRY (Teams Advancing Patient Experience: Strengthening Quality) is a program centered on the health of older adults, supported by volunteers, primary care teams, community engagement and connections, and an ICT known as the Health TAPESTRY application (TAP-App), a web-based application that supports volunteers in completing client surveys, volunteer coordinators in managing the volunteer program, and primary care teams in requesting and receiving information.

**Objective:**

This paper describes the development, evolution, and implementation of the TAP-App ICT to share the lessons learned.

**Methods:**

A case study was conducted with the TAP-App as the case and the perspectives of end users and stakeholders as the units of analysis. The data consisted of researchers’ perspectives on the TAP-App from their own experiences, as well as feedback from other stakeholders and end user groups. Data were collected through written retrospective reflection with the program manager, a specific interview with the technology lead, key emailed questions to the TAP-App developer, and viewpoints and feedback during paper drafting from other research team members. There were 2 iterations of Health TAPESTRY and the TAP-App and we focused on learnings from the second implementation (2018-2020) which was a pragmatic implementation scale-up trial using the Reach, Effectiveness, Adoption, Implementation, and Maintenance framework at 6 primary care sites across Ontario, Canada.

**Results:**

TAP-App (version 1.0), which was iteratively developed, was introduced as a tool to schedule volunteer and client visits and collect survey data using a tablet computer. TAP-App (version 2.0) was developed based on this initial experience and a desire for a program management tool that focused more on dual flow among users and provided better support for research. The themes of the lessons learned were as follows: iterative feedback is valuable; if ICT will be used for research, develop it with research in mind; prepare for challenges in the integration of ICT into the existing workflow; ask whether interoperability should be a goal; and know that technology cannot do it alone yet—the importance of human touch points.

**Conclusions:**

Health TAPESTRY is human-centered. The TAP-App does not replace these elements but rather helps enable them. Despite this shift in supporting integrated care, barriers remained to the uptake of the TAP-App that would have allowed a full flow of information between health and social settings in supporting patient care. This indicates the need for an ongoing focus on the human use of ICT in similar programs.

## Introduction

### Background

Currently, health care is provided by multiple providers across various disciplines and through different organizations. With patients receiving care from multiple people, it is crucial to have a system that can integrate providers and information horizontally and vertically across the health care system. The concept of integrated care is to deliver coordinated care that brings together services from across health and community-based social systems [1]. This type of care also aims to close the gap between health and social care [2] as it is seen as a way to bridge the gap among acute care, primary care, and community and social services [3]. Evidence shows that integration, coordination, and person-focused care are core features by which primary care achieves better population health outcomes [4]. To effectively integrate care, providers must ensure the continuity of sharing information about patients while allowing patients to remain at the center of their own care [5]. Therefore, this type of care relies on infrastructure to support the bridging of these traditionally siloed services and the centering of the patient in any intervention.

Information and communication technology (ICT) is an important enabler that supports the delivery of integrated and coordinated primary care [6,7]. ICT includes any health information technology that aids in the collection of health information and its processing, storage, and exchange [8]. There is evidence that ICT can support integrated care systems by fostering greater care efficiency and enhancing information exchange [9-11].

With the growing population of older adults in Canada and globally, the need to provide care that wraps around patients is even more important. In shifting to more integrated care, the changes that organizations make will need to be supported using all available resources. ICT can connect users across organizations and disciplines to share information. Appropriate communication to enhance these connections is critical for successful integrated care systems [5]. However, there are knowledge gaps in the literature describing the processes of introducing ICT into existing health and social care settings and in understanding how ICT changes work and information flows. Given the challenges that the introduction of anything new can present when incorporated into existing systems, careful planning and evaluation of these infrastructures are necessary.

Health TAPESTRY (Teams Advancing Patient Experience: Strengthening Quality) offers an opportunity to describe the development and evolution of ICT as part of a pragmatic complex intervention rooted in primary care. The program creates connections among trained community volunteers, interprofessional primary health care teams, novel technology, and community engagement and connections through improved system navigation [12,13]. The aim is to help older adults stay healthier for longer in the places where they live. A total of 2 evaluations have been completed through a randomized controlled trial. The study showed that patients who received Health TAPESTRY walked more (mean difference 1.13, 95% CI 0.31-1.95), had fewer hospitalizations (incidence rate ratio 0.37, 95% CI 0.18-0.77), and saw their primary care team more (mean difference 1.52, 95% CI 0.84-2.19) [12,13].

The key ICT within Health TAPESTRY is the Health TAPESTRY app (TAP-App), a web-based application that has 3 interfaces (briefly described below). In Health TAPESTRY, trained volunteers conduct visits in older adults’ homes to discuss the clients’ health and life goals, while identifying their health and health-related social needs using validated tools or surveys adapted by the research team. These surveys were administered by volunteers, facilitated by and recorded in the TAP-App on tablets through the volunteer interface. During the visits, the volunteers had the opportunity to answer client questions and provide relevant information on community programs and services that may be of interest to the client. After visits, volunteers write *social context* information on the TAP-App about their own perspectives of the client. Volunteers were also invited to write narratives on the TAP-App, which were stories of their own experiences with the potential to be used for research or program development purposes, as needed. A volunteer coordinator at each site handled the management and scheduling of volunteer and client visits through the TAP-App (ie, the volunteer coordinator interface).

The TAP-App creates an automated PDF TAP-Report that is shared with the huddle team at the patient’s primary care site through a huddle interface. The huddle is a subgroup of people within the primary care team who meet weekly to discuss with clients in the program. The huddle is composed of at least 3 providers from different disciplines (eg, physician assistant, occupational therapist, nurse, and physician). The huddle members view TAP-Reports from their own interprofessional lens and work together to create individualized plans of care for clients based on the information collected. The huddle teams are then able to document a summary of their review and plan of action on each TAP-Report through the huddle interface. They also have the option to send volunteers back to clients through the huddle action checklist, which contains a list of options for engaging volunteers in the plan of action, including discussing changes in clients’ health needs, planning the achievement of goals, or connecting clients to community-based health and social services. These recommendations are communicated to volunteers via volunteer coordinators who receive this information on the TAP-App. After volunteers follow-up with clients, they communicate any new information back to the huddles through the Follow-Up Report, another automated PDF report created by the TAP-App. A detailed description of Health TAPESTRY is in the published protocol [12].

### Objectives

In this paper, we describe the development and evolution of a specific ICT in Health TAPESTRY, the TAP-App. This story is presented as a case study to share the lessons learned during its development, evolution, and implementation.

## Methods

### Design

The case under study is the TAP-App itself, and the units of analysis are the perspectives of end users and stakeholders [14]. We used the Stake understanding of case studies [15], including a focus on qualitative results, involvement of researchers’ impressions as key sources of data, detail provided to assist in naturalistic generalization, and lack of a specific start or end time of data collection and analysis. Although our case was partly bounded by the start and end dates of the randomized controlled trial that was conducted (described elsewhere), the TAP-App went beyond these temporal boundaries; hence, we describe development and vision both before and after. The settings that bound our case were numerous and are described in the *Setting* section below.

### Data Collection

The data in this study came from the direct perspectives of key members of the research and implementation team, who are also represented as authors in this paper. The lessons learned in this paper constitute the key themes from the team’s perspectives in implementing the TAP-App in Health TAPESTRY. Specific data sources to understand how the TAP-App was implemented in its 2 phases were a written retrospective reflection from the program manager, an interview with the technology lead (facilitated by SD and JG), and key questions administered via email to the software developer.

Although the lessons learned in this paper came directly from the program team, their understanding of the implementation of the TAP-App was informed by the feedback that the team received from many other stakeholders and end users (including Health TAPESTRY clients, community volunteers, volunteer coordinators, primary care providers, and administrative or other primary care team members). This feedback came to the team via email, volunteer *lunch and learns*, volunteer narratives submitted via the TAP-App, and during verbal debriefs with volunteer coordinators. Furthermore, we gathered viewpoints and feedback from other research team members who developed and implemented the TAP-App at each of the 2 main stages. SD and JG collected this information and combined it with the direct observations of the research team to add detail and clarity to understanding the lessons learned in this work.

### Data Analysis

Although there was no traditional qualitative data set for this study’s data because of the structure of data collection, the project team members (ie, the authors and key informants) discussed lessons learned throughout and after project implementation. LL and SD developed the initial *Lessons Learned* section in this paper based on the key themes that continued to arise in these project conversations and their own perspectives as implementers. JG and SD then edited the lessons learned after collecting more information from key informants (ie, the interview with the project lead and key questions to the developer). Throughout this process, member-checking was continued, that is, feeding back the written lessons learned to the key informants, to ensure that the written representation fit their understanding of the process.

### Methods to Improve Rigor

We used several methods to enhance rigor in this case study. We worked to enhance credibility and confirmability through triangulation of various data sources (ie, respondents), data collection methods, and individual interpreters of the data [16,17]. We further enhanced credibility by prolonged engagement of respondents while ICT was used in a multi-year program [16]. Finally, although this case study focused deeply on a specific ICT, we used a thick description that may aid in understanding the potential for transferability or naturalistic generalization of the results to other settings and ICTs [15,16].

### Setting

The TAP-App was developed, hosted, and driven by an academic university department, McMaster University’s Department of Family Medicine. The department includes subgroups—2 of which are devoted to information technology and research—and were the 2 areas of the department that managed the TAP-App. During the implementation of Health TAPESTRY–Ontario (2018-2020), which is the period of focus of this study, the TAP-App was used in 6 family health teams (FHTs) across the province of Ontario, Canada to support older adult clients. FHTs are primary health care teams that bring traditional family physician–led practices together with an array of interprofessional providers such as nurses, social workers, dietitians, pharmacists, and others [18]. Interprofessional providers in an FHT may be colocated or located at different sites. The FHTs, as well as the communities they serve, vary in size, with populations ranging from 2710 to 536,917 as of the 2016 Census [19,20]. In addition to its use by FHTs, as described above, the TAP-App was used by trained community volunteers working in clients’ homes and by the organizations and individuals that supported both these volunteers and the communication exchange among elements. A national humanitarian charitable organization supported 4 of the 6 sites with 4 volunteer coordinators. A coalition of multiple agencies focused on community health supported the final 2 sites with 1 volunteer coordinator.

### Development and Evolution of the TAP-App

#### TAP-App (Version 1.0)

The first version of the TAP-App was programmed in-house at McMaster University’s Department of Family Medicine, using Java. The technology was still in active development at the start of implementation, which led to an iterative nature of development and the opportunity to integrate feedback from users. During the initial implementation of Health TAPESTRY that took place at just 1 FHT in Ontario (2014-2015), the technology was a single log-in webpage with different interfaces for volunteers and administrators (ie, volunteer coordinators and research staff) and for the 2 clinical sites within this FHT. The volunteer interface was where surveys for each client could be accessed during home visits. Upon completion, the automatically generated PDF TAP-Report was not shared with the primary care team via a huddle interface; instead, the research staff uploaded the TAP-Reports to the clients’ electronic medical records (EMRs) for the primary care team to review. Therefore, they also did not have the huddle action checklist, nor did volunteers have the follow-up visit or Follow-Up Report options, though volunteers did conduct preplanned 3-month follow-up visits which were not a part of the second implementation of Health TAPESTRY. As in the second version of the TAP-App, the volunteer coordinator interface held client and volunteer information but also had a scheduler for coordinating volunteer availability and visit times. The question data could be extracted from the TAP-App for research purposes, although it was not in the ideal format for data analysis.

#### TAP-App (Version 2.0)

Building on the initial Health TAPESTRY experience, the McMaster University Department of Family Medicine invested in a new iteration of the technology that would be supported on Research Enterprise Management of Information (REMI), a new multi-tenant software platform built using Java and Angular to facilitate collaborative research initiatives. The second large-scale implementation of Health TAPESTRY at 6 sites (2018-2020)—enabled by the TAP-App—was the first department project to use REMI, with modifications specific to the program.

**Figure 1:**
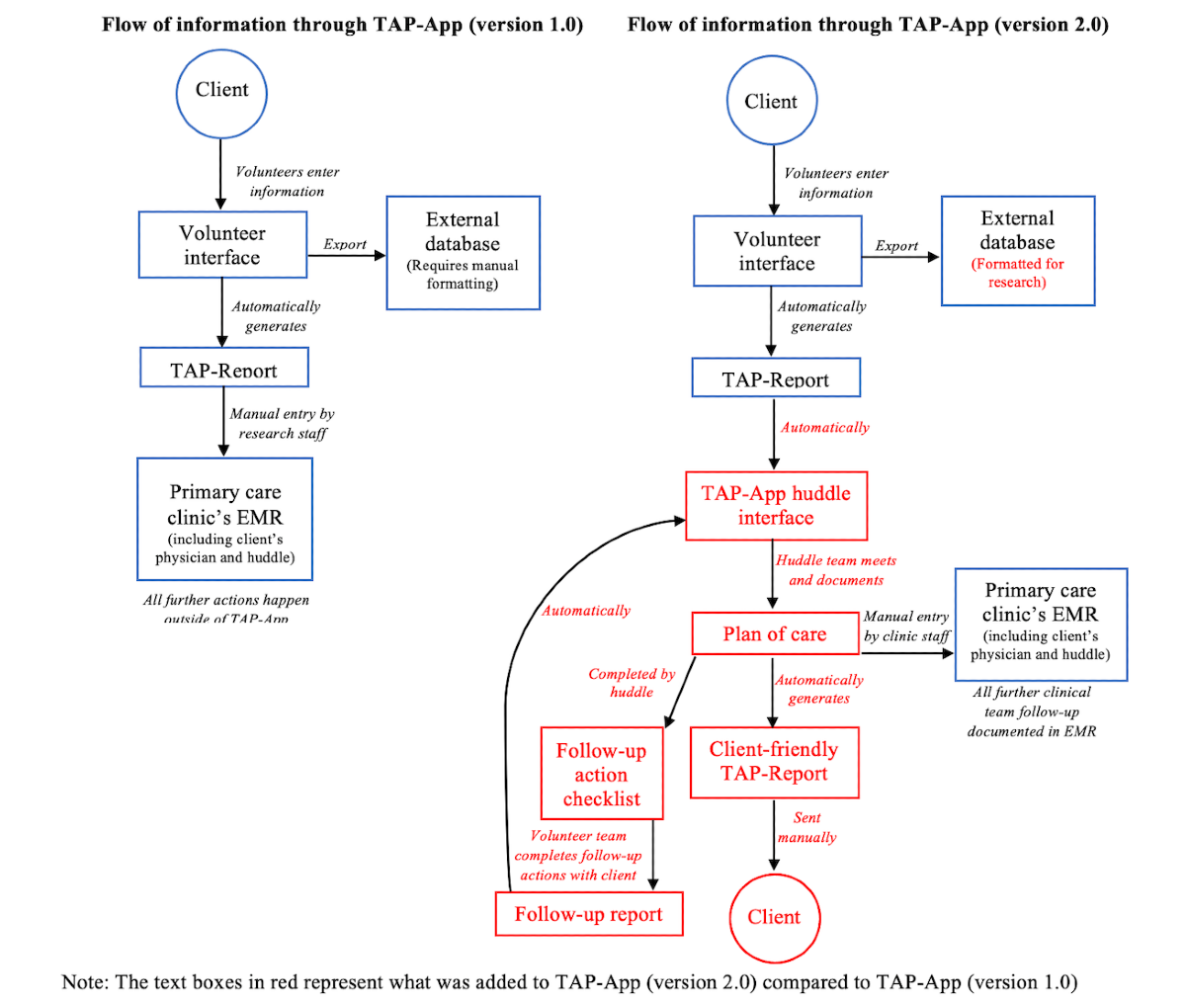
Health Teams Advancing Patient Experience: Strengthening Quality (TAPESTRY) application—TAP-App (version 1.0) versus TAP-App (version 2.0). EMR: electronic medical record; TAP-App: Health TAPESTRY app.

There were several advancements in the existing technology with the goal of establishing a 2-way versus 1-way flow of information between clients and interprofessional huddle teams (Figure 1). First, a huddle interface was created, which allowed TAP-Reports to be generated directly in the huddles. This new interface provided huddle a place where a client’s plan of care could be documented. From here on, the huddles could use the new huddle action checklist. The huddle interface also allowed huddles to create a client-friendly version of the TAP-Report, including a plan of care that could be mailed to clients. Finally, to improve the flow of information, this iteration of the TAP-App enhanced the functionality of exporting data to allow them to be uploaded to other data storage locations (eg, REDCap [Research Electronic Data Capture; Vanderbilt University] electronic capture tools) and be easily reported to sites to support quality improvement, quality assurance, or research purposes.

The design was again iterative and implementation was stepwise in nature, as rollout of the program and technology did not occur in all 6 FHTs simultaneously. Each site was able to customize its survey packages based on community or clinic needs and preferences. The TAP-App was still in active development when implementation began on the first site, which allowed the team to solicit and receive feedback from users (as described in the *Data Collection* section).

#### The Tap-App ICT: Bringing a Vision to Life

**Figure 2:**
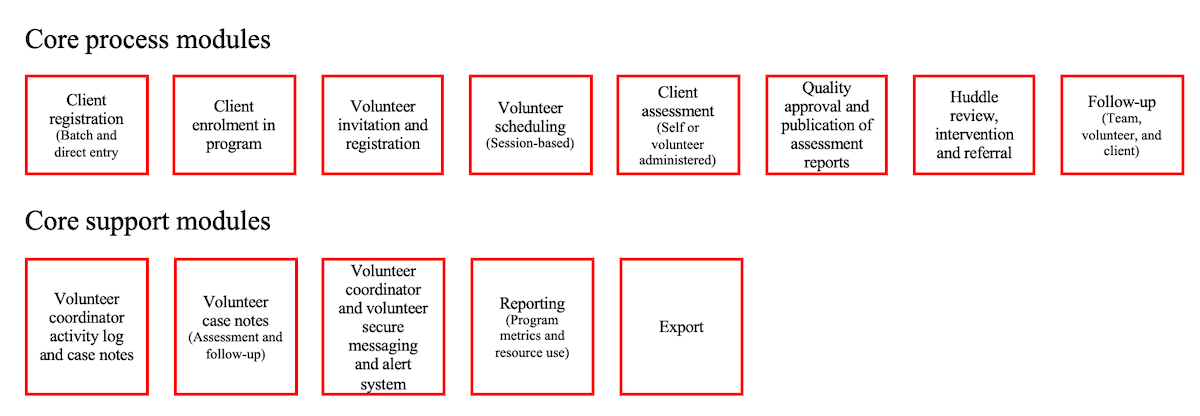
Research Enterprise Management of Information (REMI): powering the Health TAPESTRY (Teams Advancing Patient Experience: Strengthening Quality) app and more.

Although the TAP-App was first introduced as a tool to schedule volunteer and client visits and collect survey data with tablet use in focus, a broader vision underpinning this ICT was further developed during implementation. As outlined earlier, within the department, there was a desire to create a *program management tool* that could underpin all aspects of a complex intervention such as Health TAPESTRY. This shift led to the creation of a tool (the TAP-App hosted on REMI) that provided organizational support, management of clinicians and community partners, logic in data collection, and the opportunity for information to flow among users in a scalable and customizable tool that could support research and education enterprises (see Figure 2 for a detailed look). At the time of writing, the vision for the technology included some elements that had not been fully introduced, such as enhanced volunteer management and tracking and the opportunity for others in the department to use the technology.

### Contributing to Integrated Care Through the TAP-App

Models of integrated care are centered on the values of *collaborative, coordinated, comprehensive*, and *holistic care* [21]. By the end of the implementation, we were able to design a technology that made strides toward supporting these core values of integrated care through ICT. The TAP-App provided a platform that enabled communication between primary care providers and 2 community-based volunteer agencies, as well as their community volunteers, to support *collaboration* between the health and social sectors. This technology allows for the *coordination* of information within and across teams by using trained volunteers to collect data for clinicians and volunteer agencies, allowing for the expanded coordination of services for clients. The follow-up action component of the TAP-App was not extensively used by primary care teams to request volunteer support, in part because some of the items on the follow-up checklist did not mirror real-life patient needs; for example, items did not include specialist or hospital referrals as the primary care teams managed those themselves. Regardless, some clients experienced the *comprehensiveness* of having multiple providers and services across disciplines involved in their plan of care in a more integrated and less fragmented manner. Finally, this technology was embedded into a person-centered program with a focus on what mattered most to clients rather than what was the matter with clients. This introduced components of a *holistic* approach, as the TAP-App was used to support this aim and collect health as well as social, socioeconomic, emotional, and other dimensions to create a plan of care that would reflect client needs across the health and social spectrums.

## Results and Discussion

### Participants, Positionality, and Reflexivity

The authors of this paper were the participants from whom the themes of the lessons learned came. The roles they held in Health TAPESTRY were research assistant, research associate, technology (digital health) lead, program manager, research coordinator, practice model lead, software developer, data manager, executive lead, and evaluation lead. Of the 10 authors, 5 (50%) were women, 4 (40%) were men, and 1 (10%) was gender nonbinary. Of the 10 people, 7 (70%) were present in both phases of the Health TAPESTRY program, and 3 (30%) were present only in the second phase.

As developers, implementers, and evaluators of the technology under study, we had an implicit bias in the shared hope for program success. However, the 2 phases of project work allowed us to reflect on our unique position and further develop the app to overcome barriers and maximize its success. Beyond that, we aimed to take a pragmatic approach to understanding our lessons learned, considering both what worked and what did not work.

### Lessons Learned

#### Iterative Feedback Is Valuable

The introduction of any new technology will inevitably have both expected and unexpected effects on operations and workflows, even beyond the initial implementation. Therefore, continuous iterative feedback is necessary from users throughout. Given the many end users (or, in some cases, stakeholders who did not actively use the technology themselves) involved in a complex program such as Health TAPESTRY, there is a need to develop a process for managing feedback. It is also important to prioritize suggestions into high-, medium-, and low-priority categories. Some potential questions and guidelines that we found helpful—and encourage others to consider using in determining the end users’ needs—during our implementation are as follows:

1. Would this alter the mechanisms in which data moved among users?
2. Would this require retraining for end users?
3. Would this change the way in which a client experienced the program?
4. Was this outside of the scope and capacity of the technology?

A key to the successful implementation of ICT is the involvement of end users. Buntin et al [10] highlighted the *human element* being a critical component of health technology implementation, which emphasizes the importance of provider feedback and *buy-in*. As the literature has identified, it is important to plan ICT early and deliberately, with monitoring and end user involvement throughout [8]. The involvement of end users appears to be a necessary element for the successful implementation of technology [5] because the implementation of ICT will create new tasks and processes. Iterative feedback will help move the implicit experience of an end user into explicit knowledge as ICT will likely introduce new ways of working at various levels by numerous people. See Table 1 for an overview of the 5 key lessons learned from our implementation of the TAP-App.

**Table 1:** Five key domains to consider when implementing a new information and communication technology (ICT) to support integrated care.

Domain	Why is it important	When is it important
Iterative feedback	Iterative end user feedback is vital for successful implementation of an ICT as different stakeholders involved have different needs and workflows that need to be considered.	Throughout implementation
Purpose of the ICT	An ICT that will be used for research, in addition to program implementation, must consider the needs of both purposes.	Development of the ICT
Integration into existing workflow	The ICT should support and enable existing workflows to facilitate the normalization of the ICT into practice.	Throughout implementation
Interoperability	Interoperability has both advantages and disadvantages. There is a need to consider the feasibility and practicality of an ICT being interoperable with other software; it is also possible that interoperability should not be the goal.	Development of the ICT
Limitations of technology	On the basis of the program being implemented; an ICT may be unable to complete all tasks within a program or the ICT may not be the best option to complete those tasks; a human element may be required.	Development of the ICT

#### If ICT Will Be Used for Research, Develop it With Research in Mind

Computer-assisted data collection for research use has been considered acceptable and has been widely adopted for at least 25 years [22]. However, when developing an ICT with the key purpose of being part of a program (rather than a research study), the research needs to evaluate whether the program can sometimes be overlooked. This is exactly what happened within the development of TAP-App (version 1.0), which made it very difficult to manage the spreadsheet of data as the output. We identified the problem and remedied it during the development of TAP-App (version 2.0) by including an embedded researcher (LL) in the ICT development team. The only way to scale a technology that may be used for research purposes (eg, understanding pre–post changes in participant outcomes or for general program evaluation) is to have a usable downloadable file (or way to develop summary reports), ideally one that can be converted into the appropriate file types for statistical software programs. Although this is not a common key learning in the literature in this field, other authors have emphasized the importance of being aware of concurrent changes to other systems when implementing a new ICT system [23]. Although these authors referred to technology systems, it is just as important to consider any system of work, including program versus research needs.

#### Prepare for Challenges With Integration of ICT Into Existing Workflow

The integration of ICT into existing practices and workflows takes time and requires an understanding that there will be challenges until it becomes a normalized part of the regular experience. This is not a new learning on its own; the literature in the field describes how introducing new ICT introduces new ways of working, which organizations and providers may be resistant to and often, an inadequate understanding of the clinical work environment causes new ICT systems to fail [5,24,25]. We have worked to understand how to manage these barriers. Although the normalization process theory was not fully used as an underpinning for this project, we considered the elements of normalizing a new technology into the existing workflow as we implemented the TAP-App. Normalization process theory states that new interventions must interact with the service organization, practices, and ways in which providers engage with patients to be successful [26]. Along with existing work processes, implementation must interact with the existing *information ecology*, the activities in which users are already served by technology, such as the current channels of communication and storage of information [5].

Therefore, technology needs to support and enable the workflow of clinicians and not dictate it. By developing a new iteration of the TAP-App on REMI, the technology’s ability to achieve a level of flexibility and scalability allows the technology to be implemented in multiple existing contexts. However, despite including flexibility, scalability, and the human element of feedback within our development of the TAP-App in both iterations, there were still some difficult areas when fitting it into the usual practice. One of these was the log in to a secondary website by providers, that is, the use of the huddle interface. This additional step added time, already at a premium for health care providers, and an issue that can be a major constraint in introducing new ICT into practice [8].

It is also important to implement ICT that is flexible enough to change per the local context, but neither the technology nor the end users can be too rigorous in the application of the technology. Although end users need to see value in adopting a new ICT [23], its developers and implementers cannot expect too many users and users cannot expect too much ICT. Steele Gray et al [24] recommended that adopting ICT into integrated care models requires a balance between a user-focused model and disruptive innovation, as ICT will inevitably introduce new ways of working for each user. However, the literature on innovation adoption suggests that users are likely to only support components that reflect practice as usual [27], a contradiction that can be difficult to navigate. Implementation of the TAP-App resulted in similar findings, as providers ended up adopting components of the technology that were useful to them and not the parts that added work or changed the workflow.

#### Ask Whether Interoperability Should Be the Goal

In the adoption of ICT, the concept of interoperability, specifically between external software and EMRs, will likely always need to be considered and is often seen as the end goal for any new health-related technology [28]. Interoperability is “the ability of health information systems to work together within and across organizational boundaries” [29]. Although there is evidence to support the benefits of ICTs that are interoperable with EMRs [30], the TAP-App was not intended to be fully interoperable during this implementation. It was not feasible, given the sheer number of EMR systems currently in use across Canada, including the 6 sites for this study. This may be a common barrier when implementing ICTs and may make them interoperable with EMR systems. This barrier is even larger with the implementation of programs such as Health TAPESTRY, which would require information systems that spanned the health and social sectors. The lack of interoperability among systems has been considered a key barrier in other studies [24]. However, because it was not feasible to fully integrate the TAP-App into an EMR, it does not mean that more integration would not be appreciated by providers. If providers have something similar to an embedded link in their EMR that takes them only a single click to access, they may not see it as a distinct site or different from their usual workflow.

Even if we had pushed for full interoperability between the TAP-App and EMRs, it may have addressed some of the disruptions in workflow. However, it has been shown that providers need to first see a great enough value in the ICT to fully integrate it into their work. In Health TAPESTRY, the value of ICT is not only the TAP-App itself but also the program elements that the huddle can access with the TAP-App, which introduces an additional barrier to access, as the primary care team should see a great enough value in the information contained in the TAP-Report and in the work of volunteers who are following up with their patients. In observing the challenges of integrating new health ICT, Planitz et al [23] found that the adoption of health ICT relied on providers identifying the functionality of new systems and that they were reluctant to change work processes during adoption. Therefore, the consideration of how well human factors are considered in introducing new ICT, and how well the ICT’s functions are suited to end users may actually provide more value than the consideration of the interoperability of an ICT into an EMR. The human element is the key.

#### Know That Technology Cannot Do it Alone...Yet

There are inherent benefits and growing knowledge of the importance of developing human-centered ICT, including both the uptake and usability of the technology, which has been described in this case study and in other literature [31,32]. At the core of Health TAPESTRY are community volunteers administering surveys directly to clients, building rapport, and viewing clients in their own spaces, with their visits and questions facilitated by the TAP-App. This element is a benefit of the program and it would be remiss to suggest that technology could do this *instead*. Technology as the solution is not a true solution, as a human element is important in programs such as this; form should follow the function for an ICT to be successful.

Another human touch point within Health TAPESTRY was the addition of volunteer coordinators to interprofessional huddles, which further enabled the practice of integrated care, helping reach out to the community beyond the volunteer organizations themselves. While the TAP-Report to the huddle team provided necessary information, the volunteer coordinator was able to provide further narrative. In addition, this connection allowed the huddle teams to liaise directly with volunteer coordinators about possible follow-up actions for volunteers and resources to connect with patients. It also allowed the volunteer coordinator to remind the huddle teams of the community-based health and social-service options within their context and the role volunteers can play in connecting patients to these, as well as helping with any TAP-App–related issues. However, although this was an in-person activity, the volunteer coordinators’ connections to the huddles were fully internet-based. As the entire world shifted to a more internet-based environment during the COVID-19 pandemic, we have seen many fields turn to more internet-based methods of connection. Through internet-based inclusion of community-based partners in interprofessional primary care huddles, we could keep the human element of connection, using it to further humanize the use of technology [33], while still working to provide personalized, wrap-around care for clients.

### Limitations

We acknowledge several limitations to this study. First, the authors of this paper understand the development and implementation of the TAP-app were themselves the developers and implementers of this ICT. This may introduce bias. However, we were not aiming for an entirely unbiased picture. Instead, we were directly looking to further understand perspectives of the people closest to the technology. We also practiced reflexivity in understanding our position in the project. Another limitation was that there was no direct data set with actual quotes that could be used. This was a restriction of the way we set up the evaluation. Finally, while there were a great number of stakeholders who provided perspectives to the authors throughout implementation, these perspectives were filtered through authors’ understanding and positionality.

### Conclusions

Although ICT systems are used to support coordinated and integrated care through information sharing, access to care, and continuity of services [5-7,24,34], there is a limited understanding of the process of introducing ICT into existing health and social care settings [24]. We have contributed to reducing this gap in the literature by describing the development and implementation of a specific ICT, the TAP-App, within the Health TAPESTRY program. The TAP-App helped enable the human-centered elements of Health TAPESTRY. However, despite the advancements made in the design of the TAP-App, there remain barriers to achieving the uptake of technology to allow for a fully improved flow of information between health and social settings. The key lessons for introducing ICT to enhance information exchange across sectors are that a system of iterative feedback to inform the design is important and that introducing a new ICT will inevitably cause implicit and explicit changes to existing workflows. In addition, human perspectives, interactions, and relationships may be more vital than ensuring interoperability between ICTs and EMRs. Maintaining a human-centered approach to integrated care is still key, whether a human-centered approach can also be brought in through internet-based communications or other technology.

## Abbreviations

EMR: electronic medical record
FHT: family health team
ICT: information and communication technology
REDCap: Research Electronic Data Capture
REMI: Research Enterprise Management of Information
TAPESTRY: Teams Advancing Patient Experience: Strengthening Quality
TAP-App: Health TAPESTRY application
